# Effectiveness of tele-rehabilitation using AI-guided exercise and pain neuroscience education for fibromyalgia (FIBROIA): Protocol for a randomized controlled trial

**DOI:** 10.1371/journal.pone.0349017

**Published:** 2026-05-22

**Authors:** Marco Antonio Morales-Osorio, Romualdo Ordóñez-Vega, Gustavo Adolfo Alomía Penafiel, Leidy Tatiana Ordoñez-Mora

**Affiliations:** 1 Universidad San Sebastián, Facultad de Ciencias de la Rehabilitación y Calidad de Vida, Escuela de Kinesiología, Carrera de Kinesiología, Concepción, Chile; 2 Centro de Salud Familiar San Vicente, Sala de Rehabilitación Física, Talcahuano, Chile; 3 COMBA R&D Laboratory, Faculty of Engineering, Universidad Santiago de Cali, Cali, Colombia; 4 Department of Health, Physiotherapy Program, Health and Movement Research Group, Universidad Santiago de Cali, Cali, Colombia; Mayo Clinic College of Medicine and Science, UNITED STATES OF AMERICA

## Abstract

**Introduction:**

Fibromyalgia (FM) is a chronic condition characterized by widespread pain and cognitive dysfunction, with pharmacological treatments offering limited efficacy. Although Pain Neuroscience Education (PNE) and therapeutic exercise are evidence-based interventions, accessibility and adherence remain major challenges particularly in underserved regions such as Latin America. This trial investigates the effectiveness of a 12-week tele-rehabilitation program (FIBROIA) that integrates Artificial Intelligence (AI)-guided exercise with PNE to enhance access to comprehensive multimodal care.

**Methods and analysis:**

This multicentre, randomized, assessor-blinded, parallel-group controlled trial will enroll fifty adults meeting the 2016 ACR criteria for FM. Participants will be randomly assigned (1:1) to either the intervention group or enhanced usual care. The intervention consists of three personalized exercise sessions per week delivered through the Rehbody AI platform, which provides real-time biomechanical feedback, along with a weekly PNE module designed to reconceptualize pain. The primary outcome is the change in pain intensity, measured using the Visual Analogue Scale (VAS), at week 13 (±7 days), immediately following completion of the 12-week program. Secondary outcomes include the Fibromyalgia Impact Questionnaire–Revised (FIQ-R), lower-limb strength assessed by the 30-Second Sit-to-Stand test, and health-related quality of life measured with the EQ-5D-3L. Statistical analyses will follow an intention-to-treat (ITT) framework.

**Discussion:**

The FIBROIA protocol addresses the urgent need for scalable, evidence-based interventions in resource-limited settings. By combining AI-driven biomechanical feedback with cognitive reappraisal through PNE, this study seeks to reduce fear-avoidance behaviors and improve exercise adherence. This integrative approach aims to overcome the limitations of passive tele-rehabilitation by simulating asynchronous professional supervision, thereby ensuring both safety and technical precision in movement execution.

**Conclusions:**

If effective, this protocol will offer a robust, technology-enabled framework for remote FM management. The results could help establish a new clinical standard for accessible, patient-centered rehabilitation, bridging the gap between high-level evidence and real-world practice across diverse socioeconomic contexts.

**Trial registration:**

ClinicalTrials.gov NCT06672419.

## Introduction

Fibromyalgia (FM) is a chronic, complex, and disabling condition affecting approximately 2–4% of the global adult population [1]. It is characterized by widespread musculoskeletal pain, persistent fatigue, non-restorative sleep, cognitive dysfunction, and psychological comorbidities such as anxiety and depression, all contributing to substantial impairment in daily functioning and quality of life [2,3]. FM predominantly affects women, and recent epidemiological estimates suggest that more than 700,000 individuals in Latin America may be living with the condition, many of whom remain undiagnosed or receive suboptimal care [4].

The pathophysiology of FM is multifactorial, with central sensitization recognized as a principal underlying mechanism. This involves altered nociceptive signaling, impaired endogenous pain inhibition, neuroinflammatory processes, and dysfunctional cortical processing of sensory inputs [5,6]. Although pharmacological treatments such as tricyclic antidepressants, serotonin–norepinephrine reuptake inhibitors, anticonvulsants, and non-opioid analgesics are frequently prescribed, they typically yield only modest symptom relief and are often limited by adverse effects that compromise long-term adherence [7]. Consequently, current international guidelines recommend non-pharmacological interventions, including aerobic and resistance exercise and cognitive-behavioral strategies, as first-line therapies for FM [8,9].

Among these, Pain Neuroscience Education (PNE) has emerged as a promising approach that reconceptualizes pain within a biopsychosocial framework. PNE is an educational intervention designed to help individuals understand the neurophysiological and psychological mechanisms of pain, emphasizing central sensitization, neuroplasticity, and the interplay between cognitive, emotional, and perceptual processes. It aims to modify maladaptive beliefs such as catastrophizing and fear-avoidance while promoting active coping, behavioral engagement, and self-management [10,11]. Evidence from randomized controlled trials and systematic reviews supports the efficacy of PNE, particularly when combined with exercise, in reducing pain severity, enhancing physical function, and alleviating emotional distress among individuals with FM and other chronic pain conditions [12–15].

Telerehabilitation has emerged as a scalable and accessible modality for delivering rehabilitation interventions remotely via digital communication technologies. It encompasses synchronous (real-time video-based), asynchronous (app- or web-based), and hybrid models, thereby facilitating continuity of care and multidisciplinary collaboration beyond conventional clinical settings. Telerehabilitation has demonstrated benefits in improving adherence, engagement, and access to treatment for individuals who face geographical, logistical, or economic barriers to in-person care.

Recent advances in artificial intelligence (AI) and computer vision (CV) technologies have further expanded the potential of telerehabilitation by enabling automated motion tracking, remote supervision, and individualized progression of exercise programs. AI-driven algorithms can detect movement deviations, provide real-time corrective feedback, and adjust exercise intensity according to each participant’s functional capacity enhancing both safety and personalization. Although these technologies have shown promising outcomes in neurological and musculoskeletal rehabilitation, their application to fibromyalgia remains limited and underexplored [16–19].

Despite robust clinical evidence supporting non-pharmacological management, implementation across Latin American health systems continues to be hindered by workforce shortages, infrastructural limitations, geographical dispersion, and unequal access to specialized care [4]. In this context, integrating PNE with AI-assisted telerehabilitation represents an innovative, evidence-informed, and potentially scalable approach to improving FM management in resource-constrained settings.

The present study aims to evaluate the effectiveness of a 12-week telerehabilitation program integrating AI-guided exercise and PNE in reducing pain intensity and improving physical function and quality of life among adults with FM, compared with enhanced usual care. It is hypothesized that participants receiving the combined digital intervention will demonstrate significantly greater improvements in pain outcomes, functional performance, and biopsychosocial well-being than those receiving standard care.

## Methods and analysis

### Trial design and context

This study is a decentralized, assessor-blinded, parallel-group, randomized controlled trial designed to evaluate the effectiveness of a 12-week tele-rehabilitation program integrating artificial intelligence (AI)–guided exercise and Pain Neuroscience Education (PNE), compared with enhanced usual care, in adults with fibromyalgia (FM). Assessments will be conducted at baseline (Week 0) and post-intervention (Week 13 ± 7 days), immediately following completion of the 12-week program. The trial is prospectively registered at ClinicalTrials.gov (NCT06672419). Any protocol-relevant amendments (e.g., updates to masking descriptions or study setting terminology) will be reflected in the registry and documented in the final manuscript.

The trial is coordinated by Universidad San Sebastián, with international collaboration from Universidad Santiago de Cali for technical support and data management. As a fully remote study, all intervention procedures are delivered online, and participants complete the program in their own homes.

Recruitment will be conducted through clinician referral networks and community outreach across Latin America. Collaborating healthcare professionals from diverse clinical contexts (e.g., primary care and specialized pain or rheumatology services) will inform potentially eligible patients about the study and provide standardized recruitment materials. These healthcare facilities serve solely as referral sources; no study procedures are performed on site, and no identifiable patient information is shared with the research team without explicit participant consent. Interested individuals will contact the coordinating research team directly (or authorize contact) to complete eligibility screening and provide electronic informed consent.

To ensure procedural consistency, all participants will use the same digital platforms Rehbody.net and ProyectoFibroBrain.org featuring identical intervention protocols, user interfaces, and data-capture mechanisms. Study personnel (physiotherapists providing remote supervision and outcome assessors) will undergo centralized training prior to trial initiation, including review of a detailed operations manual, practical workshops, and competency certification. Inter-rater reliability will be established before participant enrollment and reassessed annually to maintain measurement consistency among assessors.

Trial conduct will be monitored centrally through scheduled coordination meetings, periodic review of digital activity logs, verification of protocol adherence, and corrective measures in the event of deviations. Outcomes will be collected via standardized procedures using validated Spanish-language instruments within the REDCap platform, ensuring harmonized data collection, coding, and secure transmission.

The trial will adhere to the SPIRIT 2013 statement [20] (including relevant updates aligned with SPIRIT 2025) and the CONSORT 2010 reporting standards [21,22] (see S1 Appendix).

### Patient and public involvement in trial design

During the planning phase, individuals with FM and representatives from patient support organizations across Latin America were consulted to capture their lived experiences, barriers to care, and perspectives on digital health interventions. Their contributions were instrumental in adapting the trial to patient needs.

A group of patients reviewed a preliminary version of the PNE manual, providing feedback that improved clarity, sequencing, and illustrative examples. Based on their input, comprehension questions were added to each module to reinforce learning. Patients also evaluated the usability of the AI-guided exercise component, leading to modifications in difficulty, pacing, and structure to enhance safety and personalization.

Concurrently, the PNE modules were reviewed by physiotherapists, clinical researchers, and pain science educators with expertise in the biopsychosocial model of chronic pain. This process ensured conceptual accuracy, pedagogical coherence, and cultural relevance. The final 12-module sequence was refined iteratively through patient and expert feedback, consistent with international standards for digital patient education [24,25,27].

Public involvement will continue throughout the trial via dissemination initiatives, including community webinars, open-access educational materials, and lay summaries. The final version of the PNE manual is publicly available online to promote equitable access to knowledge and support patient-centered learning.

### Eligibility criteria

Adults aged 18–65 years of any sex, with a confirmed clinical diagnosis of FM based on the 2016 American College of Rheumatology criteria [1], will be eligible. Participants must have reliable access to an internet-connected device (e.g., smartphone, tablet, or computer) and the ability to participate in a structured tele-rehabilitation program.

### Inclusion criteria

- Clinical diagnosis of FM according to ACR 2016 criteria.
- Age between 18 and 65 years.
- Ability to understand and communicate in Spanish.
- Access to a stable internet connection and a device capable of supporting telehealth interactions.
- Willingness and ability to complete the 12-week program.
- Provision of informed consent.

### Exclusion criteria

- Participation in another clinical trial within the past three months.
- Current pregnancy or breastfeeding.
- Presence of uncontrolled severe medical conditions (e.g., cardiac arrhythmias, severe pulmonary disease) that may limit participation.
- Physical disabilities or acute musculoskeletal injuries preventing execution of exercise routines (e.g., recent fractures, trauma).
- Severe cognitive impairment or neuropsychiatric disorder that hinders comprehension or adherence, defined as a score < 24 on the Mini-Mental State Examination, < 26 on the Montreal Cognitive Assessment, or equivalent validated screening tool.
- Severe uncorrected visual or auditory impairments interfering with digital interaction.

### Participant selection, recruitment, and consent

Participants will be identified through professional networks of the research team and outreach initiatives by collaborating centers in Latin America. Trained study personnel will verify eligibility using medical records and structured interviews.

Eligible individuals will be contacted via telephone or videoconference and provided with a digital information sheet describing study objectives, procedures, potential risks, and benefits. Informed consent approved by institutional ethics committees will be obtained electronically prior to baseline assessment or intervention initiation.

**Fig 1:**
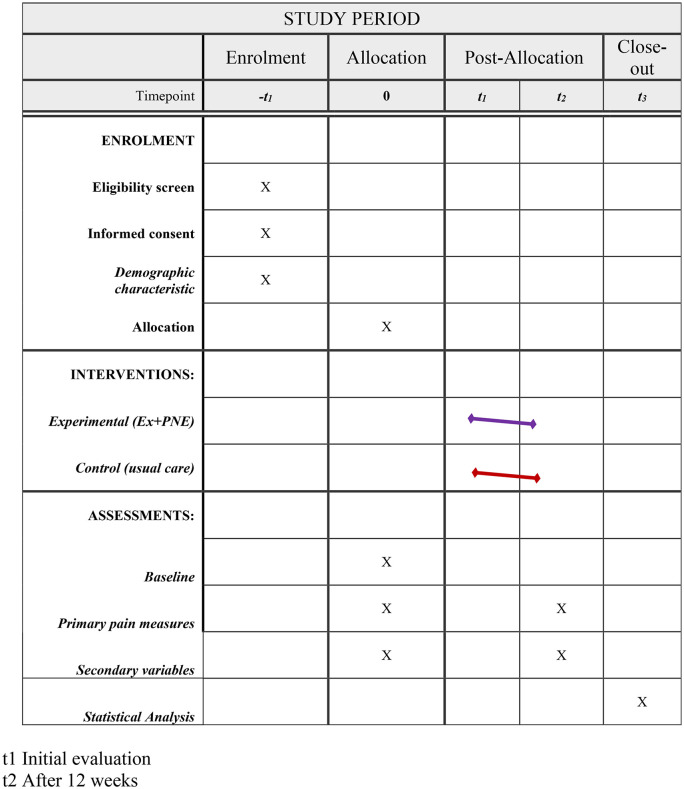
Organization according to the checklist time and interventions.

**Fig 2:**
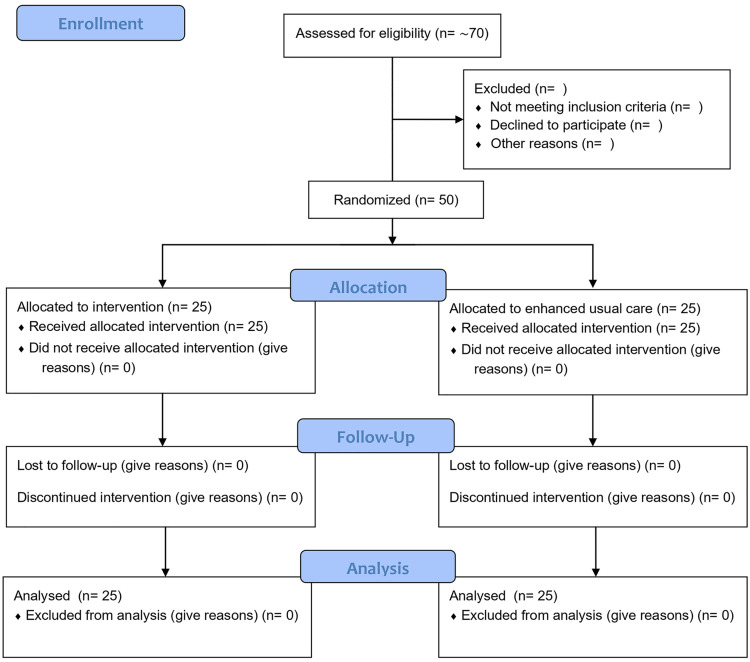
Study design. Participants (n = 50) will be randomized to either the intervention group (AI-guided tele-rehabilitation + PNE) or enhanced usual care. The intervention will last 12 weeks. Assessments will occur at baseline (Week 0) and immediately after program completion (Week 13 ± 7 days).

Following consent, participants will complete baseline demographic, clinical, and functional evaluations before randomization (Figs 1 and 2).

### Sample size

The sample size was calculated a priori to ensure both feasibility and statistical rigor. The calculation targeted the primary endpoint of pain intensity (VAS, 0–10 scale) from baseline to week 13. A minimal clinically important difference (MCID) of 2.0 cm and an expected standard deviation (SD) of 3.0 cm were assumed, based on previous randomized controlled trials in fibromyalgia populations evaluating pain reduction through behavioral and digital interventions [28,35]. Under these parameters, a total sample of 50 participants (25 per group) provides 80% power (two-tailed, α = 0.05) to detect a moderate-to-large effect size (Cohen’s d ≈ 0.65), using an independent t-test. The calculation was performed using G*Power software (version 3.1.9.6).

An attrition rate of approximately 10% was anticipated. Although higher dropout rates have been reported in some digital health trials, this estimate was considered reasonable given the structured follow-up, the short intervention period (13 weeks), and previous experience from our group showing high retention (>90%) in similar telehealth interventions for chronic pain. The sample size remains adequate after accounting for this expected loss. This calculation was finalised prior to recruitment to ensure prospective justification.

### Randomisation and allocation concealment

Randomisation will be performed using a computer-generated sequence with a 1:1 allocation ratio, employing permuted blocks of variable size (4–8 participants) to reduce allocation predictability. To minimise selection bias, randomisation will be stratified by age (<50 vs ≥ 50 years), given the established influence of age on FM outcomes.

Allocation concealment will be maintained through a centralised, web-based system (REDCap, Vanderbilt University), operated by an independent researcher not involved in intervention delivery or outcome assessment. This system provides automatic participant assignment with timestamped allocation records, ensuring transparency and traceability.

If web-based randomisation is unavailable, a validated contingency procedure will be implemented using sequentially numbered, opaque, sealed envelopes (SNOSE) prepared off-site by an independent statistician. Envelopes will be tamper-proof, opaque, and opened sequentially only after participant enrolment and baseline assessment.

### Blinding

Outcome assessors, the principal investigator, and the study statistician will remain blinded to group allocation until database lock. Randomisation will be executed centrally through the secure REDCap system. Group-specific content within the digital platforms will be activated and managed by an unblinded administrator who has no role in outcome assessment, intervention supervision, data cleaning, or statistical analysis.

Because of the nature of non-pharmacological digital exercise interventions, complete blinding of participants and treating physiotherapists is not feasible. To minimise performance bias and expectancy effects, the study employs an active comparator (sham digital experience). Participants in the control arm will access a visually identical platform interface that provides general fibromyalgia self-management education and non-specific physical activity guidance, but without the active components (AI-driven real-time biomechanical feedback and structured PNE modules).

Participants will be informed that the trial compares two digital health management approaches, without disclosing any superiority hypothesis related to the AI/PNE intervention. They will be instructed not to reveal their perceived group allocation during outcome assessments. Technical support staff and adherence monitors will not be involved in outcome data collection.

### Assessment of blinding

At the post-intervention assessment (Week 13 ± 7 days), both participants and outcome assessors will be asked to guess group allocation and to rate their confidence in that guess. Bang’s Blinding Index will be calculated to quantify the success of blinding

### Interventions

Participants allocated to the intervention arm will undertake a 12-week tele-rehabilitation program combining AI-guided exercise and Pain Neuroscience Education (PNE). This multimodal approach targets both physical and cognitive emotional dimensions of FM, aiming to promote physical activation, pain reconceptualisation, and self-efficacy [6,7,13].

### AI-guided exercise with computer vision

Participants will complete three home-based sessions per week (30–40 minutes each) via a digital platform integrating artificial intelligence and computer vision (https://rehbody.net/) Rehbody is a clinically validated telerehabilitation platform that uses the participant’s device camera to capture and analyse movement patterns through AI-driven motion tracking. The system identifies joint angles, range of motion, and posture deviations in real time, providing immediate audiovisual feedback to ensure correct technique and prevent injury. It automatically adjusts exercise difficulty based on individual performance metrics and adherence data. All data are encrypted and stored on secure servers compliant with international data protection standards (GDPR). The program follows four progressive phases: adaptation (weeks 1–3), progression (weeks 4–6), consolidation (weeks 7–9), and maintenance (weeks 10–12) [16,18]. Exercises evolve from sit-to-stand with chair support and unweighted deadlifts to glute bridges, incline push-ups, and loaded Romanian deadlifts (S2 Appendix). All sessions are remotely supervised by a licensed physiotherapist who reviews adherence reports and responds to alerts [18,23].

### Pain neuroscience education (PNE)

In parallel, Concurrently, participants will complete one asynchronous online module per week (https://proyectofibrobrain.org). The 12 sequential modules, adapted from validated frameworks [13,14,24], address topics such as nociplastic pain, central sensitisation, neuroplasticity, and psychosocial modulators [15,24,25]. Content incorporates multimedia resources, clinical metaphors (e.g., the “alarm system”), and guided reflection activities. Each module concludes with comprehension questions and self-assessment exercises to reinforce learning [26,27]. The modules were co-designed with patients with FM to ensure linguistic, cultural, and contextual relevance [13].

**Control group**Control participants will receive enhanced usual care according to clinical guidelines [3,8,9], typically including analgesics (paracetamol, tramadol), antidepressants (amitriptyline, duloxetine), anticonvulsants (pregabalin, gabapentin), and general lifestyle advice. In addition, they will use the same digital platforms with a visually identical interface and will receive standard, non-specific educational materials on fibromyalgia self-management and general physical activity guidance (without AI-guided exercise feedback or pain neuroscience education modules). Concomitant care will be documented systematically.

### Intervention fidelity and adherence monitoring

All physiotherapists will complete a standardized training program and adhere to a detailed intervention manual specifying session structure, therapeutic content, and delivery protocols. Intervention fidelity will be evaluated monthly by an independent investigator uninvolved in intervention delivery. Audits will include digital log reviews (frequency, duration, accuracy), random checks of therapist documentation, and verification of protocol compliance.

Protocol adherence will be deemed satisfactory when ≥90% of planned components are delivered as outlined in the manual. Identified deviations will trigger corrective actions such as targeted feedback, refresher training, and additional supervision until compliance is restored. Fidelity indicators—including adherence rates, frequency of deviations, and corrective measures—will be summarised descriptively and reported in the Results section to document intervention integrity [38].

Participant adherence will be monitored automatically through the digital platforms. Participants will be considered adherent if they complete ≥80% of prescribed sessions (≥29/36 exercise sessions and ≥10/12 PNE modules). Automated SMS or email reminders will be issued for missed sessions, and non-adherent participants will receive motivational follow-up from the research team to address barriers.

Control group participants’ engagement (login frequency, material access, and time spent on the platform) will be monitored equivalently to ensure comparable exposure time. Study staff will conduct monthly check-ins to verify participation and reduce attrition bias across groups.

### Discontinuation criteria

Participants may discontinue the intervention if they experience a serious adverse event (SAE), clinically significant deterioration, voluntary withdrawal, or persistent non-adherence (>30% missed sessions). For moderate adverse symptoms, exercise intensity may be adjusted, or sessions temporarily suspended. All modifications will be recorded. Participants who discontinue the intervention will be encouraged to complete follow-up assessments.

### Adverse event monitoring and reporting

All adverse events (AEs) occurring from enrolment to final assessment will be recorded, regardless of their relationship to the intervention. AEs are defined as any untoward medical occurrence, while SAEs include events resulting in death, life-threatening conditions, hospitalisation, or persistent disability.

Participants will be instructed to report any new symptoms or health changes. The digital platforms (Rehbody.net and ProyectoFibroBrain.org) automatically generate alerts when sessions are incomplete due to reported symptoms, prompting physiotherapist follow-up.

All AEs and SAEs will be documented in case report forms, detailing onset, duration, severity, relatedness, and actions taken. SAEs will be reported to the ethics committee within 24 hours. Safety data will be summarised descriptively by group in the final report, following CONSORT recommendations.

### Permitted and prohibited co-interventions

Participants in both groups may continue pharmacological treatments prescribed by their primary care providers, including analgesics, antidepressants, or anticonvulsants commonly used in FM management. They will be instructed not to initiate any new structured physical therapy, exercise program, or cognitive behavioural therapy during the trial. Engagement in such interventions outside the study protocol will be considered a protocol deviation.

At each assessment point, participants will report any additional treatments, medications, or health-related changes. These data will be documented and included as potential covariates in subsequent analyses.

### Outcomes

Outcome selection followed the IMMPACT recommendations for chronic pain clinical trials [28] and was tailored to the characteristics of FM and the digital delivery format (Table 1).

**Table 1:** Description of the measurements.

Variable Category	Variable	Dimension	Indicator	Value
**Sociodemographic Data**	Age	Chronological age	Number of complete years	18 to 65 years
	Gender	Gender identification	Participant-reported gender	Male / Female
	Educational level	Formal schooling level	Highest level completed	Primary / Secondary / Technical / University / Postgraduate
	Residence location	Geographical context	Living area	Urban / Rural
	Access to technology	Digital inclusion	Availability of internet-connected devices	Yes / No
	Employment status	Work situation	Current working status	Employed / Unemployed / Informal / Retired
**Clinical Data**	Diagnosis duration	Time since FM diagnosis	Time elapsed since official diagnosis	Numerical (months)
	Pharmacological treatment	Medication type and dose	Use of prescribed drugs	Analgesics, antidepressants, anticonvulsants (type, dose, frequency)
	Comorbidities	Associated diseases	Presence of additional chronic conditions	Yes / No (specify if relevant)
**Primary Outcomes**	Pain intensity	Subjective pain perception	Visual Analogue Scale (VAS)	0 (no pain) to 10 (worst imaginable pain)
**Secondary Outcomes**	Impact of FM	Functional limitation and symptom severity	Total score of the FIQ-R	0 to 100 (higher scores indicate worse impact)
	Lower limb function	Strength and endurance	30-Second Sit-to-Stand Test	Number of repetitions in 30 seconds
	Quality of life	Health-related QoL	EQ-5D index	−0.59 to 1.0 (higher = better QoL)
	Anxiety	Psychological distress	Numeric Rating Scale for Anxiety (NRS-A)	0 (no anxiety) to 10 (worst possible anxiety)
	Adherence barriers	Perceived obstacles to treatment	Treatment Adherence Barriers Questionnaire	Categorized: logistical, motivational, contextual, etc.

Table note: Abbreviations: FM = Fibromyalgia; VAS = Visual Analogue Scale; FIQ-R = Revised Fibromyalgia Impact Questionnaire; EQ-5D = EuroQol-5 Dimensions; NRS-A = Numeric Rating Scale for Anxiety.

### Primary outcome

The primary endpoint is change in pain intensity (VAS) between baseline (week 0) and post‑intervention at week 13 (±7 days), immediately after completion of the 12‑week programme. Secondary endpoints will be assessed at the same timepoints [29]. Pain intensity was chosen as the primary outcome because it is the cardinal symptom of FM and the most consistently recommended endpoint in chronic pain trials [28].

### Secondary outcomes

Secondary endpoints will provide complementary information on physical, psychosocial, and quality-of-life domains:

- Impact of fibromyalgia (FIQ-R; 0–100, higher scores = greater impact) The Spanish version has demonstrated high internal consistency (Cronbach’s α ≥ 0.91) and acceptable test-retest reliability (ICC ≥ 0.70 for total score) [30,31]
- Lower limb strength and endurance (30-Second Sit-to-Stand Test, validated for remote FM assessment) [32]
- Health-related quality of life (EQ-5D-3L, index −0.59 to 1, higher = better) [33]
- Psychological well-being (NRS-Anxiety; 0–10, higher = greater anxiety) [34]
- Exploratory outcome: Perceived barriers to adherence, assessed with the Treatment Adherence Barriers Questionnaire (adapted for telerehabilitation) [35]

All outcomes will be assessed at baseline (week 0) and post-intervention in week 13 (±7 days). No long-term follow-up is planned. Instruments were selected based on prior validation for remote administration, relevance to FM, and consistency with outcome domains recommended for digital rehabilitation trials [28,36].

### Multiplicity

As pain intensity (VAS) is the sole primary endpoint, secondary outcomes will be considered exploratory. No formal multiplicity adjustment will be applied; effect estimates will be reported with 95% confidence intervals to aid interpretation.

### Data analysis

Analyses will follow both intention-to-treat (ITT) and per-protocol (PP) principles, consistent with CONSORT recommendations [21]. The ITT population will include all randomised participants, analysed according to their allocated group. Missing outcome data will be addressed using multiple imputation, assuming data are missing at random. Sensitivity analyses using last observation carried forward (LOCF) and complete-case approaches will be performed to assess the robustness of the findings.

The PP population will include participants who complete at least 80% of the prescribed intervention dose (≥29/36 exercise sessions and ≥10/12 PNE modules) and have no major protocol deviations. Baseline characteristics will be summarised descriptively and compared between groups using parametric (independent t-test) or non-parametric (Mann–Whitney U test) methods, depending on normality assessed via the Shapiro–Wilk test.

The primary analysis will evaluate the change in pain intensity (VAS, 0–10 cm) from baseline to week 13 (±7 days) between groups using both independent t-tests and Mann–Whitney U tests to ensure consistency of results. The anticipated effect size (Cohen’s d ≈ 0.65) was derived from prior trials of multidisciplinary and digital interventions in fibromyalgia, which reported moderate-to-large effects on pain reduction (range 0.5–0.7) [28,36,37]. Within-group changes will be assessed using paired t-tests or Wilcoxon signed-rank tests, as appropriate. Effect sizes (Cohen’s d) with 95% confidence intervals will be reported and interpreted using conventional thresholds [38].

Secondary outcomes (FIQ-R, 30-Second Sit-to-Stand, EQ-5D, NRS-Anxiety, adherence barriers) will be analysed using similar methods. Minimal clinically important differences (MCIDs) will be defined a priori as follows: 14% for FIQ-R total score, ≥ 2 repetitions for the 30-Second Sit-to-Stand, and ≥0.07 for EQ-5D index, based on previous validation studies. For repeated measures, mixed-effects models will account for within-subject correlations and time effects [39,40]. Multivariable regression analyses will adjust for relevant covariates (e.g., baseline pain, age, pharmacological treatment).

Pre-specified subgroup analyses will explore treatment effects according to baseline pain severity (VAS ≥ 7 vs. < 7), age group (<50 vs. ≥ 50 years), and pharmacological treatment status (yes/no). Secondary outcomes will be considered exploratory. No formal multiplicity adjustment will be applied; effect estimates will be reported with 95% confidence intervals to support interpretation

All statistical tests will be two-tailed with α = 0.05. Analyses will be performed using IBM SPSS Statistics v28.0 and R software v4.3.2. Results will be presented with effect sizes, confidence intervals, and graphical summaries.

### Data security and management

All study data will be managed in accordance with the principles of Good Clinical Practice (GCP), the General Data Protection Regulation, the Health Insurance Portability and Accountability Act, and applicable national and institutional ethical standards. Each participant will be assigned a unique alphanumeric identifier to ensure anonymisation, with the master code list stored separately in a password-protected, access-restricted file available only to the principal investigator.

Data entry and storage will be performed using encrypted, password-protected digital platforms (REDCap and Rehbody), both hosted on secure institutional servers with controlled access. Double-entry verification and periodic quality audits will ensure data accuracy and completeness, with any discrepancies reconciled against source documentation.

Protocol deviations, missing values, and data inconsistencies will be systematically documented and resolved according to standardised data management procedures. Regular backups will be performed on secure servers, and study records will be retained for the legally mandated period before secure destruction in compliance with institutional policy.

### Test monitoring

A preliminary pilot phase will be conducted prior to full trial implementation to assess the clarity, duration, and feasibility of outcome instruments, as well as the usability and technical performance of the digital platforms. Findings from this phase will inform refinement of study procedures and identification of potential barriers to participant engagement.

Simultaneously, all educational and multimedia materials used in the Pain Neuroscience Education (PNE) modules will undergo expert and patient review to ensure linguistic clarity, cultural relevance, and alignment with lived patient experiences. Feedback from these evaluations will be incorporated prior to full trial rollout.

### Human ethics and consent to participate declarations

This study protocol was approved by the Comité Ético Científico of Universidad San Sebastián, Chile (Acta No. 149−24, 10 October 2024). No participants have been recruited at the time of submission. Written informed consent will be obtained from all participants prior to enrollment in the study.

### Ethics and dissemination

The study complies with the principles of the Declaration of Helsinki (2013 revision), the International Conference on Harmonisation Good Clinical Practice (ICH-GCP) guidelines, and relevant national regulations.

Prior to enrolment, all participants will receive a digital information sheet describing the objectives, procedures, potential risks, and expected benefits of the study. Written informed consent will be obtained electronically before initiating any study-related procedures. Participants will be informed that their participation is voluntary and that they may withdraw at any time without affecting their usual care.

### Confidentiality

To ensure data confidentiality, participants will be identified solely by unique alphanumeric codes. Personal identifiers will be stored separately in encrypted, password-protected files accessible only to the principal investigator. Data management procedures will comply with GDPR, HIPAA, and applicable local data protection legislation.

### Dissemination plan

All participants will receive ongoing feedback throughout the intervention and a summary of the aggregate study results. If the intervention demonstrates clinical benefit, participants in the control arm will be offered access to the program after trial completion to ensure equitable benefit distribution.

Study findings will be disseminated through peer-reviewed journals, international conference presentations, and plain-language summaries for patient advocacy organisations. The educational content used in the intervention co-designed and pre-tested with patients will be made openly accessible. Public dissemination activities, including webinars, workshops, and community forums, will further engage healthcare stakeholders, patient groups, and the broader public.

### Protocol amendments

Any substantial modifications to the protocol (e.g., changes in eligibility criteria, outcome measures, or analytical strategy) will be submitted for review by the Comité Ético Científico of Universidad San Sebastián and updated in the ClinicalTrials.gov entry. All modifications will be transparently reported in the final publication.

### Data monitoring and auditing

Given he minimal-risk nature of the intervention (exercise and education), a full independent Data Monitoring Committee (DMC) will not be convened. Instead, an independent safety monitor external to the study team and with expertise in rehabilitation and clinical research will periodically review safety data, including adverse events and protocol deviations, to ensure participant protection.

Safety and adherence data will also be reviewed monthly by the supervising physiotherapists and the principal investigator. Serious adverse events will be reported immediately to the ethics committee and documented according to institutional and regulatory requirements.

Although external audits are not planned, the study may be subject to internal audits by the sponsoring institution, Universidad Santiago de Cali, or by the ethics committee if required. All monitoring and audit reports will be archived and made available upon request.

### Compensation for harms

The intervention is classified as low risk, comprising supervised exercise and educational components. Nonetheless, all adverse events potentially related to study participation will be recorded and reported to the ethics committee. Participants experiencing study-related harm will receive appropriate medical care at no personal cost. No financial compensation will be provided. The sponsoring institution (Universidad Santiago de Cali) maintains insurance coverage for clinical research activities, including medical treatment for study-related injury.

### Dissemination of results

Results will be reported in accordance with the CONSORT recommendations [21]. Findings will be submitted for publication in peer-reviewed journals and presented at international scientific conferences. Summaries will also be shared with patient organisations, primary care teams, and regional rehabilitation networks throughout Latin America to promote clinical translation and public health integration.

## Discussion

The FIBROIA trial represents an important advance in the management of fibromyalgia (FM), a chronic nociplastic pain disorder characterised by central sensitisation and limited responsiveness to pharmacological monotherapy [8]. By integrating Pain Neuroscience Education (PNE) with AI-guided therapeutic exercise, this protocol introduces a scalable, multimodal rehabilitation strategy specifically designed for populations with limited access to specialised care.

Growing clinical evidence supports the synergistic benefits of combining PNE with exercise as the gold standard for non-pharmacological FM management [8,10]. Although aerobic and resistance training are among the most effective interventions, their implementation is often impeded by high attrition, kinesiophobia, and fear-avoidance behaviours. Within this framework, PNE functions as a cognitive-behavioural foundation that enables patients to reinterpret pain not as a sign of tissue injury but as a reflection of neural hypersensitivity [41]. This cognitive reappraisal fosters self-efficacy and therapeutic engagement, both critical to sustained exercise adherence.

Integration of the Rehbody platform represents a significant evolution from traditional, passive tele-rehabilitation models. Standard digital interventions typically rely on static instructional media and lack the corrective feedback necessary for biomechanical precision. In contrast, AI-enabled real-time feedback simulates therapist supervision, providing asynchronous yet interactive oversight that enhances patient confidence, safety, and adherence [42]. This dynamic, feedback-driven approach also allows for personalised progression of exercise intensity, addressing the heterogeneity of FM symptomatology.

From a public-health perspective, the scalability of the FIBROIA protocol holds particular relevance for low- and middle-income regions such as Latin America. By reducing logistical and financial barriers associated with frequent in-person visits, this model democratizes access to multidisciplinary, evidence-based care [42]. If proven effective, the combined AI + PNE framework may redefine remote rehabilitation standards, establishing a cost-effective, patient-centred approach that bridges the gap between high-level clinical evidence and real-world practice [20].

## Conclusions

The FIBROIA protocol provides a comprehensive, technology-enabled framework for addressing the multidimensional challenges of fibromyalgia. By integrating neuroscience-based education with AI-assisted exercise supervision, this trial has the potential to establish a new benchmark for accessible, patient-centred, and scalable rehabilitation strategies in chronic pain management.

### Strengths and limitations of this study

- This study presents an innovative tele-rehabilitation program that integrates artificial intelligence guided exercise and Pain Neuroscience Education (PNE), providing a novel, patient-centered approach to fibromyalgia (FM) management.
- The fully remote design enhances accessibility and scalability, particularly benefiting underserved and rural populations.
- The use of validated and widely recognized outcome measures ensures methodological rigor and facilitates comparison with existing research.
- The relatively small sample size may limit the generalizability of the findings.
- Blinding of participants, outcome assessors, and statistical analysis strengthens internal validity; however, potential variability in adherence to digital interventions may introduce bias.

## Supporting information

S1 Appendix SPIRIT 2025 checklist of items to address in a randomized trial protocol. (DOCX)

S2 Appendix Description of the protocol. (DOCX)
