# Supplementary material for: Effectiveness of tele-rehabilitation using AI-guided exercise and pain neuroscience education for fibromyalgia (FIBROIA): Protocol for a randomized controlled trial
Source: PLoS One. 2026 May 22;21(5):e0349017. doi: 10.1371/journal.pone.0349017 (PMC13196953; doi:10.1371/journal.pone.0349017)
Supplement: S2 Appendix — (DOCX) [file pone.0349017.s002.docx]

**Appendix 2. Protocol Effectiveness of Tele-Rehabilitation Using AI-Guided Exercise and Pain Neuroscience Education for Fibromyalgia (FibroIA): A Randomized Controlled Trial**

# 1. Administrative Information

Trial Title: Effectiveness of Tele-Rehabilitation Using AI-Guided Exercise and Pain Neuroscience Education for Fibromyalgia (FibroIA): Protocol for a Randomized Controlled Trial

Trial Acronym: FibroIA

Trial Registration: ClinicalTrials.gov Identifier: NCT06672419

Protocol Version: v1.0 – August 20, 2025 v2.0 – December 27, 2025
Sponsor: Universidad Santiago de Cali (Call No. 01-2026)

Collaborating Institutions: Universidad San Sebastián (Chile); Centro de Salud Familiar San Vicente (Chile); Instituto Nacional de Cardiología Dr Ignacio Chávez (Mexico); Centro de Artritis y Osteoporosis (Colombia)

Principal Investigator: Leidy Tatiana Ordóñez-Mora, PhD (Universidad Santiago de Cali, Colombia)

Ethics Approval: Comité Ético Científico, Universidad San Sebastián, Chile (Acta No. 149-24, 10 October 2024)

Funding: This research has been funded by Dirección General de Investigaciones of Universidad Santiago de Cali under call No. DGI-01-2026. The sponsor has no role in study design, data collection, analysis, or publication decisions.

# 2. Introduction

Background and Rationale: Fibromyalgia (FM) is a chronic and disabling condition characterized by widespread pain, fatigue, poor sleep, and cognitive impairments, affecting 2–4% of adults worldwide. Pharmacological treatments provide limited benefit. International guidelines recommend non-pharmacological interventions, such as exercise and cognitive-behavioural strategies. Pain neuroscience education (PNE) and exercise have shown efficacy, and AI-supported tele-rehabilitation may enhance accessibility.

Objectives: Primary Objective: To evaluate the effectiveness of a 12-week tele-rehabilitation program combining AI-guided exercise and PNE in reducing pain intensity compared to usual care. Secondary Objectives: To assess effects on functional capacity, fibromyalgia impact, health-related quality of life, psychological well-being, and adherence barriers.

# 3. Methods: Participants, Interventions and Outcomes

Study Design: Multicentre, randomized, double-blind, parallel-group controlled trial. Duration: 12 weeks. Assessments at baseline (week 0) and post-intervention (week 13 ±7 days).

Study Setting: Participants recruited from Chile, Colombia, and Mexico.

Eligibility Criteria: Inclusion – Adults 18–65, fibromyalgia diagnosis (ACR 2016), internet access, Spanish literacy. Exclusion – Pregnancy, severe uncontrolled conditions, disability preventing exercise, participation in another trial within 3 months.

Interventions: Experimental arm (AI-guided exercise + PNE): 3 weekly sessions (30–40 min) via Rehbody.net, progressive exercises, and 12 online modules (ProyectoFibroBrain.org). Control arm: Usual care (pharmacological management + lifestyle advice).

Outcomes: Primary – Pain intensity (VAS). Secondary – FIQ-R, 30-Second Sit-to-Stand Test, EQ-5D-3L, NRS-Anxiety, adherence barriers questionnaire.

Timeline: The intervention will last 12 weeks. Assessments will occur at baseline (Week 0) and immediately after program completion (Week 13 ± 7 days).

# 4. Methods: Assignment of Interventions

Randomisation: Computer-generated 1:1 with permuted blocks (4–8), stratified by age (<50 vs ≥50). Managed via REDCap; fallback: sealed envelopes.

Blinding: Participants, investigators, outcome assessors, and statisticians blinded. Emergency unblinding only in medical necessity.

# 5. Methods: Data Collection, Management and Analysis

Data Collection: Standardised validated instruments in Spanish.

Data Management: Unique participant IDs, encrypted servers, GDPR/HIPAA compliant, regular audits.

Statistical Analysis: Primary – VAS change analysed with t-test/Mann–Whitney U, effect size (Cohen’s d). Secondary – FIQ-R, Sit-to-Stand, EQ-5D, NRS-A, adherence barriers analysed with mixed models/regression. Analyses by intention-to-treat and per-protocol.

# 6. Methods: Monitoring

Adverse Events: All AEs recorded; SAEs reported within 24h to ethics committee.

Auditing: No DMC; internal monitoring by PI and ethics committee.

Compensation: Care through sponsor insurance; no monetary compensation.

# 7. Ethics and Dissemination

Ethics: Approved by Comité Ético Científico, Universidad San Sebastián (Acta No. 149-24).

Confidentiality: Anonymised codes, GDPR/HIPAA compliance.

Dissemination Plan: Results shared in journals, conferences, patient organisations. Control participants offered program post-trial.
